# HappyMaps: a single hub of resources on children and young people's mental health for parents and professionals

**DOI:** 10.1192/bjo.2021.125

**Published:** 2021-06-18

**Authors:** Georgina Griffiths, Jasmin Krischer, Cara Roberts-Collins, Elaine Lunts

**Affiliations:** 1 Avon and Wiltshire Partnership Trust; 2 General practitioner; 3 Royal United Hospitals Bath NHS Foundation Trust

## Abstract

**Aims:**

Mental health issues in children and young people are a growing concern and the benefits of intervening early are well established for many mental health problems, but existing Child and Adolescent Mental Health Services (CAMHS) are often over-stretched with variable waiting times for assessment. Many children also have problems which do not reach the referral thresholds and parents are left to find advice elsewhere. Existing resources for parents are scattered across many different websites and therefore difficult to access both for parents and professionals working with young people. With this in mind, and in consultation with CAMHS Bristol and many other stake-holders (including parents themselves) we designed an easily navigable website intended as a single comprehensive portal of resources for parents of children with mental illness and difficulties.

**Method:**

Qualitative research methods were used to gather information about how the website should be designed and also to gather feedback once the website was live. Focus groups were performed with parents/carers and stakeholder discussions took place to inform the design of the website. Once the website was live, surveys via a Survey Monkey link on the website and Google Analytics were used to evaluate the website.

**Result:**

60,000 users have utilised the website since the launch in March 2019. Two thirds of users are women and one third are men. Most popular webpages that are visited are primary, secondary, help-in -a-crisis and self-help for young people. Positive feedback has been collected from both parents/carers and service providers. The website has continued to develop and is now a registered charity and has received community lottery funding, which will allow for further evaluation and developments.

**Conclusion:**

HappyMaps has been successful in providing a single hub of information for parents/carers, GPs, CAMHS workers and teachers. Future work involves evaluating the website and attracting interest from other CAMHS teams and professionals in other areas of the UK so that they can create HappyMaps sections for their populations.

